# NRGAMTE: Neurophysiological Residual Gated Attention Multimodal Transformer Encoder for Sleep Disorder Detection

**DOI:** 10.3390/brainsci15090985

**Published:** 2025-09-13

**Authors:** Jayapoorani Subramaniam, Aruna Mogarala Guruvaya, Anupama Vijaykumar, Puttamadappa Chaluve Gowda

**Affiliations:** 1 Department of Electronics and Communication Engineering, Sri Shanmugha College of Engineering and Technology, Salem 637304, India; jayapoorani.ece@shanmugha.edu.in; 2 Department of Artificial Intelligence and Machine Learning, Dayanand Sagar College of Engineering, Bangalore 560111, India; aruna-aiml@dayanandasagar.edu (A.M.G.); anupama-aiml@dayanandasagar.edu (A.V.); 3 Department of Electronics and Communication Engineering, Dayanand Sagar University, Bangalore 560111, India

**Keywords:** cross-modal interactions, modality-wise residual gated cross-attention fusion, neurophysiological residual gated attention multimodal transformer encoder, one-dimensional convolutional neural network, physiological signals, sleep disorder

## Abstract

Background/Objective: Sleep is significant for human mental and physical health. Sleep disorders represent a crucial risk to human health, and a large portion of the world population suffers from them. The efficient identification of sleep disorders is significant for effective treatment. However, the precise and automatic detection of sleep disorders remains challenging due to the inter-subject variability, overlapping symptoms, and reliance on single-modality physiological signals. Methods: To address these challenges, a Neurophysiological Residual Gated Attention Multimodal Transformer Encoder (NRGAMTE) model was developed for robust sleep disorder detection using multimodal physiological signals, including Electroencephalogram (EEG), Electromyogram (EMG), and Electrooculogram (EOG). Initially, raw signals are segmented into 30-s windows and processed to capture the significant time- and frequency-domain features. Every modality is independently embedded by a One-Dimensional Convolutional Neural Network (1D-CNN), which preserves signal-specific characteristics. A Modality-wise Residual Gated Cross-Attention Fusion (MRGCAF) mechanism is introduced to select significant cross-modal interactions, while the learnable residual path ensures that the most relevant features are retained during the gating process. Results: The developed NRGAMTE model achieved an accuracy of 94.51% on the Sleep-EDF expanded dataset and 99.64% on the Cyclic Alternating Pattern (CAP Sleep database), significantly outperforming the existing single- and multimodal algorithms in terms of robustness and computational efficiency. Conclusions: The results shows that NRGAMTE obtains high performance across multiple datasets, significantly improving detection accuracy. This demonstrated their potential as a reliable tool for clinical sleep disorder detection.

## 1. Introduction

Sleep is an important physiological need for all individuals, as it refreshes and restores the human body [1,2,3]. Moreover, both the quantity and quality of sleep are essential for maintaining a healthy life [4,5]. However, various kinds of sleep disorders, such as Insomnia (INS), sleep apnea, Rapid Eye Movement (REM) sleep Behavior Disorder (RBD), and Periodic Leg Movement (PLM), disrupt sleep quality [6,7,8]. Sleep disorders increase the risk of negative health conditions, including reduced immunity, impaired cognitive function, daytime sleepiness, cardiovascular problems, and headaches [9,10,11]. As the number of people suffering from sleep disorders continue to rise, accurate diagnosis through sleep monitoring has become essential [12,13,14,15].

Sleep-related features are detected using Electroencephalogram (EEG) time waveforms, which are recorded to diagnose patients [16]. The characteristics of various sleep stages are also reflected in other signals, such as Electromyograms (EMGs) or Electrocardiograms (ECGs), recorded continuously to support diagnostic decisions [17,18]. Conventional sleep detection involves the manual evaluation of time waveforms, with records spanning several hours of sleep [19]. Highly trained experts analyze the features that occur in various phases of sleep and associate them with specific sleep pathologies. However, this procedure is time-consuming and costly [20,21].

Recent advancements in Machine Learning (ML) algorithms have made the automatic scoring of sleep increasingly feasible [22,23]. Several ML algorithms for recognizing EEG patterns have been developed, but multimodal algorithms that integrate various signals are still limited [24,25,26]. Although manual sleep scoring shows clear advantages when multiple modalities are used, EEG alone introduces ambiguity. A more reliable diagnosis can be achieved by analyzing the behavior of multiple simultaneously recorded signals [27].

Multimodal diagnostic systems are designed to address issues related to modeling and representing various data modalities, as well as the arbitration among diagnostic results produced by different modalities [28,29,30]. In such systems, modalities are processed through parallel channels, and fusion occurs in the final decision-making phase [31]. Each processing channel independently represents the data and applies specific model architectures [32]. The integrated feature extraction phase is fed into a single method for the final diagnosis [33].

Transformer-based models are particularly suitable for addressing these drawbacks [34]. They have demonstrated high potential in computer vision, with techniques such as path encoding and shifted windows proving effective in capturing spatial data [35].

Recently, transformer-based architectures such as LGSleepNet [8] and BiTS-SleepNet [13] have shown promising results in sleep analysis.

The proposed Neurophysiological Residual Gated Attention Multimodal Transformer Encoder (NRGAMTE) model introduces both architectural and functional novelties. Unlike Core-Sleep, which lacks explicit residual paths in attention fusion, NRGAMTE incorporates a Modality-wise Residual Gated Cross-Attention Fusion (MRGCAF) mechanism. This mechanism selectively filters noisy interactions while preserving essential signal-specific features through learnable residual paths. BiTS-SleepNet is designed for single-modality EEG data and cannot model cross-modal interactions. LGSleepNet emphasizes local–global temporal modeling but does not integrate modality-aware embeddings or gated attention fusion. Unlike previous models that convert EEG, EOG, and EMG signals into spectrograms or images for processing with Two-Dimensional Convolutional Neural Networks (2D-CNNs), this research retains the 1D sequential form. This approach avoids information loss during transformation, reduces the computational complexity, and enables a more natural multimodal fusion.

The aim of NRGAMTE is to demonstrate that raw 1D modeling, combined with gated attention and transformer integration, achieves competitive performance. In the proposed framework, each modality is independently processed by One-Dimensional Convolutional Neural Networks (1D-CNNs) and fused through residual gated attention, ensuring superior interpretability and robustness.

### 1.1. Problem Statement

Sleep disorder detection is a challenging task due to the high inter-subject variability in sleep patterns and overlapping symptoms among disorders such as PLM, narcolepsy, REM behavior disorder, and insomnia. Relying on a single physiological signal fails to capture the complete spectrum of disorders, which introduces ambiguity in diagnosis and limits generalization across different patient populations. Furthermore, overlapping features among disorders require methods capable of learning fine-grained distinctions across modalities.

### 1.2. Objective

The main objective of this work is to develop a precise and computationally efficient Deep Learning (DL)-based framework for sleep disorder detection using multimodal physiological signals. The algorithm segments raw sleep data into 30-s epochs, enabling a consistent temporal analysis. For each segment, informative time- and frequency-domain features are extracted to represent different physiological patterns across EEG, EMG, and EOG signals. Each modality is independently encoded using 1D-CNNs, thereby preserving signal-specific characteristics.

For the efficient integration of multimodal features, a modality-wise residual gated cross-attention fusion mechanism is incorporated. This mechanism filters redundant information and integrates relevant data across modalities. With the addition of a learnable residual path, information loss during gating is prevented, and model stability during training is enhanced.

### 1.3. Contributions

The key contributions of this article are as follows:

- We developed a sleep disorder detection framework that combines EEG, EMG, and EOG signals to capture complex physiological information and address the limitations of the unimodal approaches;
- We employed a fixed 30-s windowing approach with both time- and frequency-domain features to effectively differentiate between various sleep disorders;
- We utilized separate 1D-CNNs for each modality to preserve signal-specific characteristics and learn robust high-level feature representations;
- We designed a multimodal transformer encoder that captures long-range temporal dependencies while retaining modality-specific features through learnable residual paths, achieving superior accuracy across multiple sleep disorder classes;
- We proposed the MRGCAF mechanism to perform cross-attention among modalities, filtering noisy and redundant interactions via a forget gate, while the learnable residual path preserves critical information during gating. This improves interpretability and detection performance.

The remainder of this article is organized as follows: Section 2 analyzes existing algorithms along with their advantages and limitations. Section 3 describes the dataset. Section 4 details the proposed NRGAMTE algorithm. Section 5 presents the model validation and results. Finally, Section 6 concludes the article.

## 2. Literature Review

This section highlights various ML and DL algorithms for sleep disorder detection, focusing on both single-modality and multi-modality approaches.

### 2.1. Single-Modality-Based Algorithms

Hui Wen Loh et al. [36] developed a DL algorithm based on a 1D-CNN for CAP detection and homogenous three-class sleep classification, including wakefulness (W), REM, and NREM sleep, using standardized EEG recordings.

Manish Sharma et al. [37] presented an ML approach that automatically classified ECG signals into sleep disorder classes. CAP data were used, including Polysomnography (PSG) recordings from individuals with and without sleep disorders. A Wavelet scattering network was employed to extract features from ECG signals, and various classifiers were evaluated. Among them, the ensemble bag-of-trees classifier achieved optimal performance.

Megha Agarwal and Amit Singhal [38] introduced a device for differentiating between the A and B stages of sleep. Small EEG segments were with Gaussian filters to acquire sub-band elements. Features were extracted using statistical measures, and a minimum-redundancy–maximum-relevance test identified the most significant features.

Barproda Halder et al. [39] implemented a multi-resolution deep neural network with temporal and channel attention to detect A-phases and their subtypes. A multi-branch 1D-CNN was applied with each branch using different kernel sizes to capture the features at various frequency resolutions. An attention-enabled transformer network was used for dynamic temporal relationships among CAP features derived from single-channel EEG data.

Aditya Wadichar et al. [40] developed a hierarchical algorithm for sleep disorder detection and CAP stage classification using single-channel EEG recordings. The algorithm classified the CAP sequence as healthy or unhealthy and further identified specific disorders in unhealthy cases, including PLM, REM, RBD, Nocturnal Frontal Lobe Epilepsy (NFLE), Narcolepsy, and insomnia. A hybrid network combining Long Short-Term Memory (LSTM) and CNN was employed.

Kamlesh Kumar et al. [41] proposed a CNN-enabled approach for insomnia detection using EEG signals, avoiding the need for complex PSG. Morlet-wavelet-based continuous wavelet transforms and the Smoothed Pseudo-Wigner–Ville Distribution (SPWVD) were applied to generate EEG scalograms. Convolutional layers were then used for feature extraction and image classification. The Morlet transform provided effective time-frequency distribution. A Morlet-wavelet-based CNN (MWTCNNNet) was introduced for classifying healthy versus insomnia patients.

Keling Fei et al. [42] suggested an automatic sleep-staging algorithm to improve sleep disorder detection and treatment. Due to the weak EEG signal features and complex frequency elements during sleep stage transitions, a Wavelet-based Adaptive Spectrogram Reconstruction (WASR) method with seed growth was used to extract time-frequency features. The Teager energy operator was integrated into WASR to capture dynamic EEG features, generating additional spectrograms. These spectrograms were processed by a lightweight CNN with depth-wise separable convolutions, leading to effective sleep stage detection and enhanced EEG feature representation.

### 2.2. Multi-Modal Based Methods

Jiquan Wang et al. [43] proposed CareSleepNet, a hybrid DL network for automatic sleep staging using PSG recordings. A multi-scale convolutional-transformer epoch encoder was designed to extract local and global features from each sleep epoch. A cross-modality context encoder based on co-attention modeled the relationships among modalities, while a transformer-based sequence encoder captured the temporal dependencies across epochs. Finally, learned feature representations were input into an epoch-level classifier to determine the sleep stages.

Yi-Hsuan Cheng et al. [44] developed a distribution neural network for multimodal sleep stage identification, offering competitive results with relatively low computational and data requirements. The system employed an ensemble of independent CNN-based classifiers, each trained on a single-modality signal, with a fully connected shallow neural network integrating the results.

Yi-Hsuan Cheng et al. [45] further proposed the Multimodal and Multilabel Decision-Making System (MML-DMS). This framework integrated multiple classifier algorithms, including CNNs and shallow perceptrons, where each module processed a distinct modality and label. The information flow among modules supported the final detection of both sleep stages and disorders. The fusion of multilabel and multimodal features significantly enhanced the diagnostic performance compared with the single-label and single-modality algorithms.

From the above analysis, single-modality algorithms such as 1D-CNN, LSTM, and wavelet-based CNNs have demonstrated effectiveness in detecting specific sleep disorders. However, they suffer from limited generalization, difficulty in handling overlapping disorder characteristics, and reduced performance when the signal quality is compromised. Moreover, several existing algorithms fail to adequately integrate multimodal signals, and are essential for capturing the complex physiological interactions in sleep disorders.

This study addresses these limitations by developing a multimodal method that combines EEG, EOG, and EMG signals through a MRGCAF mechanism. The proposed NRGMTE enhances detection accuracy, robustness, and interpretability by learning modality-specific characteristics and capturing the temporal dependencies across signal patterns.

Table 1 presents a comparison of the existing sleep disorder detection methods with the proposed NRGAMTE model in terms of the dataset, modality, methodology, and performance.

### 2.3. Model Selection

The developed model includes a 1D-CNN with a multimodal transformer-based encoder for efficiently capturing temporal and modality-specific features. The 1D-CNN was chosen for its ability to extract hierarchical features from signals such as EEG, EMG, and EOG with minimal computational overhead. This CNN preserves the essential waveform characteristics for each modality.

To address the limitations in handling long sequences, a multimodal transformer-based encoder was incorporated for temporal modeling. Moreover, an MRGCAF mechanism was integrated to improve the fusion of multimodal features by dynamically filtering irrelevant cross-modality interactions and highlighting discriminative patterns. The inclusion of a learnable residual path prevents information loss during the gating process. By developing this model, we obtain superior accuracy, interpretability, and robustness in intra- and inter-subject interactions.

## 3. Dataset

This section describes the datasets used in this article, namely, the CAP Sleep Database and the Sleep-EDF expanded dataset.

### 3.1. CAP Sleep Database

The CAP sleep database [46], publicly available through PhysioNet, contains 108 PSG recordings collected at the Sleep Disorders Center. The dataset includes recordings from subjects with varying health conditions: 16 healthy individuals, 5 with narcolepsy, 9 with insomnia, 22 with RBD, 10 with PLM, 2 with bruxism, 4 with Sleep-Disordered Breathing (SDB), and 40 with NFLE. The cohort consists of 42 females and 66 males, aged 14–82 years.

Each recording spans 9–10 h and includes 3 or more EEG signals, EOG, chin and tibial EMG, respiratory, airflow, ECG, and Arterial Oxygen Saturation (SaO_2_). For consistency, the data were selected based on sampling frequency and acquisition channel. The chosen subset includes EEG, ECG, EOG, and EMG signals sampled at 512 Hz.

Because the number of recordings for SDB and bruxism was too small, and many contained abnormal or missing data, these classes were excluded. In total, 75 subjects across six classes—PLM, RBD, healthy, narcolepsy, insomnia, and NFLE—were retained. All records were segmented into 30-s epochs based on sleep annotations, resulting in 77,374 epochs. Excluding bruxism and SDB ensured statistical reliability and prevented skewed model learning.

In this study, the analysis focuses on six classes: PLM, RBD, insomnia, narcolepsy, NFLE, and healthy. Table 2 presents the dataset description of the CAP Sleep Database.

### 3.2. Sleep-EDF Expanded Dataset

The Sleep-EDF expanded dataset [47], also publicly available through PhysioNet, contains 197 whole-night sleep recordings. The dataset includes EEG, EOG, chin EMG, and manual sleep stage annotations. The EEG was recorded at the Fpz-Cz and Pz-Oz positions.

The sampling frequencies are as follows: EOG and EEG at 100 Hz, EMG at 1 Hz in the Sleep Cassette (SC) subset, and EMG at 100 Hz in the Sleep Telemetry (ST) subset. To construct input sequences, raw EEG, EOG, and EMG signals were segmented into non-overlapping 30-s epochs, following the American Academy of Sleep Medicine (AASM) standards.

To prevent data leakage, complete subject recordings were assigned exclusively to either the training, validation, or test set. This ensured that no epochs from the same subject appeared in more than one split, thereby preserving the integrity of the performance evaluation and preventing data bleed across splits.

## 4. Proposed Method

**Figure 1:**
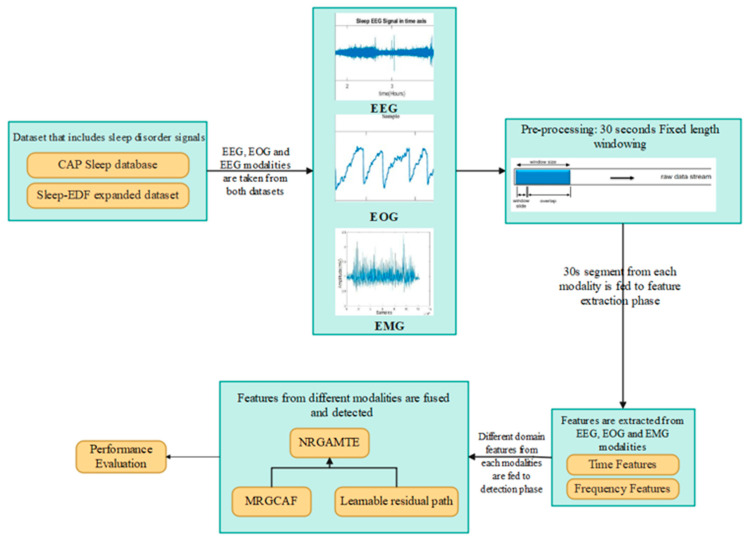
Workflow of proposed NRGAMTE-based framework for multimodal sleep disorder detection.

For accurate and interpretable sleep disorder detection, the proposed framework employs a multimodal transformer architecture combined with a modality-wise residual gated attention fusion mechanism. Each physiological signal (EEG, EMG, and EOG) is transformed into the same embedding space using a 1D-CNN, which preserves the modality-specific temporal features. These embeddings are processed by a gated attention mechanism that learns the relevance of each modality. A learnable residual path is included in the gating mechanism for preventing data loss caused by aggressive gating; this residual connection preserves and reweights modality features to enables feature fusion for final detection. Figure 1 shows the workflow of the proposed NRGAMTE-based sleep disorder detection framework.

### 4.1. Pre-Processing Using Fixed-Length Windowing

Fixed-length windowing is used to split continuous signals into 30-s epochs; each epoch is treated as one sample. The 30-s window [48] is the standard length used in sleep staging and clinical analysis, which ensures temporal consistency across samples. Fixed 30-s epochs also reduce computational complexity and enable time-localized feature extraction. A 30-s epoch provides a balance between temporal resolution (to detect subtle disorders) and sufficient data for meaningful feature extraction. Very short windows can lead to noisy features and reduced robustness in spectral attributes, while very long windows may span multiple sleep-stage transitions, reduce label clarity, and temporally smooth or mask abnormal patterns. Low-frequency bands require longer windows to be represented accurately, whereas short windows may fail to capture low-frequency content correctly.

### 4.2. Feature Extraction

Preprocessed signals are fed to the feature extraction phase, where time- and frequency-domain features are extracted to help differentiate sleep disorders.

#### 4.2.1. Time-Domain Features

- Mean is the average amplitude of the signal in a 30-s window; it provides information about the signal’s activity level. Abnormal events like arousals or bruxism can shift the mean amplitude due to muscle activation or cortical excitation.
- Standard Deviation (SD) measures variability of the signal amplitude around its mean. High SD indicates abrupt fluctuations that are present during arousals or movement. Disorders such as sleep apnea can increase signal variance because of frequent interruptions.
- Skewness quantifies asymmetry of the signal distribution; skewed signals indicate nonuniform bursts. This helps distinguish stable sleep from disordered sleep.
- Kurtosis measures the presence of outliers in the signal. High kurtosis indicates sharp spikes or transients; transient events are relevant for identifying sleep stages and disorders like narcolepsy.
- Zero Crossing Rate (ZCR) counts the number of times the signal crosses the zero-amplitude line and reflects rapid changes in the time domain. Higher ZCR indicates higher frequency activity. EMG signals during movement disorders typically show high ZCR, whereas deep-sleep EEG shows lower ZCR due to slow-wave dominance.

#### 4.2.2. Frequency-Domain Features

##### Fast Fourier Transform (FFT)

FFT is the engineering realization technique of the Discrete Fourier Transform (DFT) [49]. In accordance with the features of the rotation factor, the DFT solution and calculation process are described. Mathematical expression for DFT is given in Equation (1):

(1) $$X\left(k\right)=\sum_{n=0}^{N-1}x\left(n\right)e^{-j(2\pi /N)kn},0\leq k\leq N-1$$

In Equation (1), $X\left(n\right)$ denotes the discrete physiological signal within one 30-s epoch of length N samples, and $X\left(k\right)$ denotes its DFT. The computation requires $N^{2}$ complex multiplications and $N\left(N-1\right)$ complex additions and its mathematical expression is given in Equation (2):

(2) $$X\left(k\right)=\sum_{n=0}^{N-1}x\left(n\right)\omega_{N}^{kn}$$

From the above Equation (2), ω denotes the rotation factor. The time-domain signal is represented as $x\left(n\right),\;n=1,\;2,\;\ldots ,\;N-1$. This signal is separated into two phases based on the index, and its mathematical expression is provided in Equation (3):

(3) $$\left\{\begin{array}{c} f_{even}\left(n\right)=x\left(2n\right)\;\;\;\;\;\;\\ f_{odd}\left(n\right)=x\left(2n+1\right) \end{array}\right.$$

In this stage, the index range is $n=0,\;1,\;\ldots ,\;\frac{N}{2}-1$ and its mathematical expression is shown in Equation (4):

(4) $$x\left(k\right)=\sum_{n=even}x\left(n\right)\omega_{N}^{kn}+\sum_{n-odd}x\left(n\right)\omega_{N}^{kn},n=0,1,\ldots N-1$$

In accordance with rotation scaling factor, further conversion is expressed in Equation (5):

(5) $$x\left(k\right)=\sum_{m=0}^{(N/2)-1}f_{even}\left(m\right)\omega_{N/2}^{k.m}+\omega_{N}^{k}\sum_{m=0}^{(N/2)-1}f_{odd}\left(m\right)\omega_{N/2}^{k.m}$$

The above equation is expressed as (6):

(6) $$x\left(k\right)=F_{even}\left(k\right)+\omega_{N}^{k}F_{odd}\left(k\right),k=0,1,\ldots ,N-1$$

In the above Equation (6), the $F_{even}\left(k\right)$ represents the result of index input, and the $F_{odd}\left(k\right)$ represents the result of odd index input.

##### Features Extracted from FFT

- Spectral centroid is the center of mass of the power spectrum; higher centroid indicates greater high-frequency content which is typically wake or REM. Spectral centroid helps quantify shifts in dominant frequencies across sleep stages.
- Spectral Entropy measures disorder in the power spectrum; high spectral entropy is a more complex signal with multiple active frequencies (wake and REM tend to show higher entropy than deep sleep). This feature helps detect stage instability and arousals.
- Spectral Band Power measures energy within EEG bands such as delta, theta, alpha, and beta, and reflects brain functional states during sleep. Disorders such as apnea or insomnia can produce abnormal band-power distribution; this feature assists in detecting such abnormalities.

Time- and frequency-domain features are not concatenated directly with raw signals before convolution. Instead, raw EEG, EOG, and EMG sequences are independently encoded into temporal embeddings using 1D-CNNs. Handcrafted features are processed in parallel and concatenated with CNN-derived embeddings at the representation level so that both learned and handcrafted descriptors contribute to the multimodal transformer.

### 4.3. Modality Embedding Using 1D-CNN

A standard 1D-CNN is applied to process EEG, EMG, and EOG signals independently because of its simplicity, low parameter count, and ability to capture temporal dependencies in physiological signals with low computational overhead. Compared with more complex modules such as dilated CNNs or inception, 1D-CNNs are easier to optimize and to combine with attention-based fusion models.

The modality-embedding layer transforms the extracted feature vectors for each physiological signal modality into a shared embedding space of fixed dimensionality, enabling alignment and comparison across modalities prior to fusion. Let $X_{m}\in R^{T\times F}$ denote the time-frequency features for modality across T segments with F features per segment. Each modality’s 30-s segmented features are fed to a modality-specific 1D-CNN. The modality embedding is shown through mathematical expression in Equation (7):

(7) $$Z_{m}=ReLU\left(Conv1D\left(X_{m}\right)\right)\in R^{T\times d}$$

In Equation (7), $Z_{m}$ denotes the modality embedding of fixed size d; the convolution learns localized filters from features and *ReLU* introduced nonlinearity. The resulting modality-specific latent representation $Z_{m}$ is aligned in a fixed size and format across modalities and is provided as input to the multimodal transformer encoder. This yields a compact, learnable representation for each modality and reduces overfitting by learning filters over short sequences.

Depending on the dataset sampling rate, each raw physiological signal produces a specific number of samples per 30-s epoch. For example, when sampled at 100 Hz, a 30-s window yields 3000 samples. The 1D-CNN encoder applies temporal convolution and downsampling (stride = 2 and kernel = 5) to produce a sequence of 128 tokens per modality, where each token is a 64-dimensional embedding, resulting in an output tensor of shape 128 × 64 for each modality. This tokenized representation preserves temporal granularity and is used as input to the transformer-based fusion module.

### 4.4. Multimodal Fusion and Detection Using MRGCAF

The modality embeddings are fused by the MRGCAF mechanism. MRGCAF selects informative cross-modal interactions while preserving significant features via a learnable residual path. The fused representation is provided to a transformer encoder that captures long-range temporal dependencies across sleep epochs [50]. Finally, the result is aggregated and fed to a classification layer to predict the presence and type of sleep disorder.

**Figure 2:**
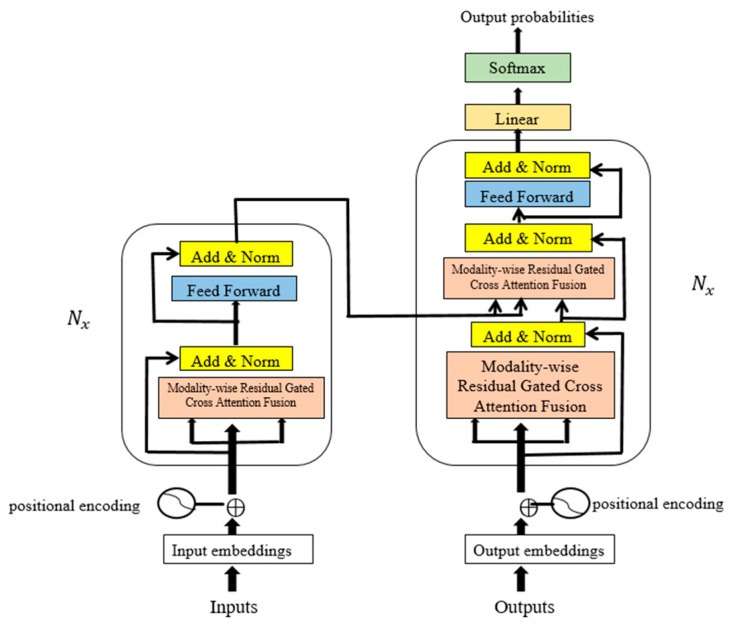
Overall architecture of NGRMTE model, including modality-specific 1D-CNN embeddings, MRGCAF fusion module, and final detection layer.

Unlike traditional multimodal transformers that rely on passive attention or simple concatenation, MRGCAF introduces a trainable fusion mechanism. A cross-modality forget gate dynamically filters redundant interactions, while the learnable residual path adaptively reweights retained features. Together, these components form gated residual attention that allows the model to learn appropriate signal-retention strategies across modality pairs. Figure 2 shows the architecture of the NRGMTE model.

#### 4.4.1. Cross-Modality Attention

The sequences from all modalities have a size of 128 × 64 and are used in pairwise cross-modality attention. The transformer receives these sequences as temporal tokens and maintains time alignment across modalities. Positional embeddings are included to preserve sequence ordering. In the NRGAMTE method, learnable positional embeddings are used to encode the temporal ordering of sequential tokens derived from EEG, EOG, and EMG modalities.

Each 30-s segment is tokenized into 128 temporal tokens of dimension 64 using 1D-CNN embedding. To preserve sequence data, a learnable positional embedding vector of the same dimension is added to every token before feeding it to the transformer encoder. This enables the model to learn relevant temporal relationships adaptively, rather than relying on fixed sinusoidal encodings. Additionally, shared positional embeddings are applied across modalities to ensure temporal alignment of cross-model tokens, while modality-specific embeddings preserve signal-specific characteristics.

This attention mechanism extracts interaction signals across modalities. Each pair of modalities is treated as one input pair (EEG–EOG, EOG–EMG, and EEG–EMG). The set of modality pairs is defined as $P=\left\{\left(eeg,eog\right),\left(eog,emg\right)\left(eeg,emg\right)\right\}$. The inputs of the interaction feature generator are modality pairs represented as $z_{\left(i,j\right),\left(i,j\right)\in P}$. Cross-modality attention of modality pair is transmitted using scaled dot-product, and the resulting cross-modality attention features are denoted by $A={\left\{a_{\left(i,j\right)}\right\}}_{\left(i,j\right)\in P}$.

#### 4.4.2. Cross-Modality Forget Gate

Cross-attention maps enable the model to extract interactions across modalities. However, $a_{\left(i,j\right)}$ of modality pair $\left(i,j\right)$ may include noise and redundant data. Such redundancy obscures useful interaction signals and reduces the effectiveness of detection. To address this, a cross-modality forget gate is introduced to filter out redundant data and preserve significant cross-modal signals.

The forget gate receives the generated cross-modality attention and processes it through a forget cell to generate filtered cross-modality features. Specifically, the cross-modality attention map of a modality pair first produces a forget vector, which regulates data flow retained for detection tasks.

Mathematical expression for forget vector of modality pair is given as Equation (8):

(8) $$f_{\left(i,j\right)}=\sigma \left(\left[a_{\left(i,j\right)}⊕z_{j}\right]W^{f}+b^{f}\right)$$

The filtered features of modality pair are then measured using Equation (9):

(9) $$h_{\left(i,j\right)}=ReLU\left(z_{i}+\left(a_{\left(i,j\right)}W^{m}+b^{m}\right)⊙f_{\left(i,j\right)}\right)$$

In Equations (8) and (9), ⊙ denotes element-wise product, ⊕ denotes concatenation, and $W^{f},\;W^{m},\;b^{f},\;b^{m}$ are trainable parameters. The original feature $z_{i}$ is preserved to enhance the actual modality, inspired by residual connection architecture.

**Figure 3:**
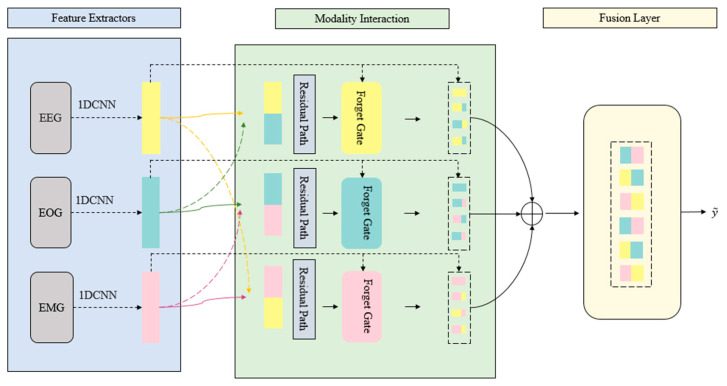
Internal architecture of the MTGCAF module. This architecture shows how modality pairs interact through gating, concatenation, and learnable residual paths.

Figure 3 shows the architecture of the MRGCAF layer. Multimodal attributes are captured from their respective modalities, cross-modality interactions are measured in modality pairs, and the resulting features are processed by the fusion layer followed by a fully connected layer.

#### 4.4.3. Fusion Layer

The output of the forget gate contains two parts: 1. actual modality features and 2. filtered cross-modality interactions. For each of the three modality pairs, the results are denoted as $H={\left\{h_{ij}\right\}}_{i,j\in v,a}^{i\neq j}$.

These are stacked across the three pairs, and cross-modality gated attention is applied to refine the representation in the cross-modality vector space. Here, $d_{h}$ denotes the dimensionality of each cross-modality attribute generated by the forget gate.

#### 4.4.4. Learnable Residual Path for Preventing Information Loss

Cross-modality gated attention effectively filters out noise and redundant interactions. However, this gating process may also suppress subtle informative signals. To address this issue, a learnable residual path is incorporated to preserve modality-specific features that could otherwise be reduced during gating process.

Unlike conventional residual connections itsimply forward the inputs asfixed, the learnable residual path adaptively reweights and retains features through trainable parameters. This enables the model to selectively preserve and amplify essential signal context. This design improves gradient flow and fusion stability. Additionally, enabling cross-model feature fusion, the developed gating mechanism plays a crucial role in improving long-range temporal dependency modeling. Moreover, the gating mechanism regulates the contribution of each modality at each fusion stage, suppressing redundant activations while propagating salient features.

This selective information stabilizes feature representations across extended temporal horizons and ensures that the transformer backbone receives contextually relevant inputs. As a result, the transformer can more efficiently capture long-range temporal dependencies while avoiding dilution of essential signals that occurs when all modalities contribute uniformly.

**Figure 4:**
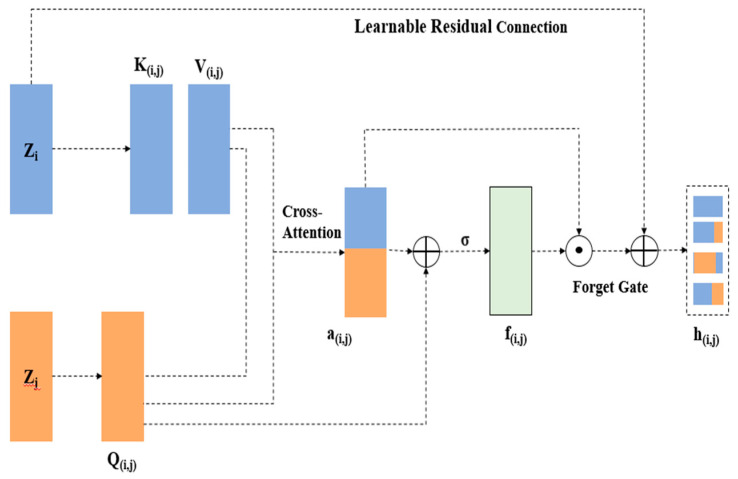
The gated cross-modality interaction architecture with one of three modality pairs.

Figure 4 illustrates the gated cross-modality interaction structure with one of the three modality pairs.

This residual path ensures robust gradient flow and protects essential features from being lost during training. The mathematical expression for the fused feature output of a modality pair is given in Equation (10):

(10) $$F_{ij}^{fused}=G_{ij}⊙{\tilde{F}}_{ij}+\left(1-G_{ij}\right)⊙F_{i}+R\left(F_{i},{\tilde{F}}_{ij}\right)$$

In Equation (10), $G_{ij}$ represents the forget gate for the modality pair, $F_{i}$ represents the actual modality feature, ${\tilde{F}}_{ij}$ represents the filtered cross-modality features from each modality, and $R\left(F_{i},{\tilde{F}}_{ij}\right)$ denotes the learnable residual path function. This dynamic learnable residual path helps the model retain significant signal content and improves training stability and performance in modality fusion.

#### 4.4.5. Model Training

In the final phase, the result of the cross-modality residual gated attention module, which contains high-level global features, is passed through two linear layers with residual connections and a SoftMax non-linear activation function to compute prediction possibilities. The mathematical expressions are given in Equations (11) and (12):

(11) $$\tilde{Z}=Z_{F}+\zeta_{\rho }\left(Z_{F}\right)$$

(12) $$P=softmax\left(\zeta_{\beta }\left(\tilde{Z}\right)\right)$$

In Equations (11) and (12), $\zeta_{\rho }$ and $\zeta_{\beta }$ represent the two linear layers with parameter sets ρ and β, and P denotes the prediction probability for each class. To minimize class imbalance caused by biased information distribution and varied detection complexity, a multi-class focal loss function is adopted. Its mathematical expression is given in Equation (13):

(13) $$FL\left(y,p\right)=-\sum_{c=1}^{C}\alpha_{c}y_{c}{\left(1-p_{c}\right)}^{\gamma }\log\left(p_{c}\right)$$

In Equation (13), C represents the total number of classes, $y_{c}$ and $p_{c}$ denote the true label and predicted possibility of class, and $\alpha_{c}$ and γ represent the balancing parameter (which handles trade-off between positive and negative instances) and the focusing parameter (which down-weights contributions from well-classified instances). In this manuscript, focal loss is used to address class imbalance in multi-class classification.

The two primary parameters of focal loss are tuned using a grid search strategy on the validation set. The optimal results are obtained with γ=2 and α=0.25, which emphasize harder-to-classify samples without causing instability. Focal loss is chosen as the training objective to address the class imbalance inherent in sleep disorder datasets. Unlike standard cross-entropy, which equally weights all instances, focal loss applies a scaling factor that reduces the influence of easily classified majority samples and highlights minority-class samples. This process is crucial in this manuscript, where disorders such as insomnia, narcolepsy, and REM-related disturbances are unrepresented compared with healthy or N2 classes. By addressing class imbalance and overlapping features, focal loss improves the model’s ability to learn discriminative features for minority disorders and enhances robustness across heterogeneous subjects.

Handling the class imbalance–sleep disorder dataset iwth general phases such as the N2 class dominating minority disorders such as insomnia and narcolepsy. To address this, focal loss is employed, as expressed in Equation (14):

(14) $$L_{FL}\left(p_{t}\right)=-\alpha_{t}{\left(1-p_{t}\right)}^{\gamma }\log\left(p_{t}\right)$$

In Equation (14), $p_{t}$ denotes the predicted probability of the true class, $\alpha_{t}$ denotes the class balancing factor, and γ is the focusing parameter. The modulating factor ${\left(1-p_{t}\right)}^{\gamma }$ down-weights well-classified majority-class instances and dynamically emphasizes minority-class instances. The proposed model allocates more learning capacity to underrepresented disorders, improving recall without requiring explicit data re-sampling. Algorithm 1 illustrates the process of the proposed NGRMTE model for reproducibility.
**Algorithm 1** Process of proposed NGRMTE model for reproducibility.Input—Raw physiological signals (EEG, EMG, EOG) Output—Detected sleep disorder class Pre-processing For each modality in (EEG, EMG, EOG)  Segment signals into 30-s epochs Feature Extraction For each 30-s segment in each modality  Extract time-domain features such as Mean, Standard deviation, Skewness, Kurtosis, and ZCR Extract frequency-domain features using FFT, such as Spectral centroid, Spectral entropy, and Spectral band power using Equations (1)–(6). Modality Embedding using 1D-CNN For each modality  Input extracted features into modality-specific 1D-CNN using Equation (7)  Obtain modality-specific embeddings (fixed dimension) Modality-wise Residual Gated Cross-Attention Fusion (MRGCAF) For each modality pair (EEG-EOG, EEG-EMG, EOG-EMG)  Compute cross-attention between modality embeddings  Apply the forget gate to filter redundant or noisy interactions using Equations (8) and (9)  Add a learnable residual path to preserve essential signal features  Fuse actual modality and filtered cross-modality features using Equation (10) Multi-Modal Transformer  Stack fused features from all modality pairs  Apply feed-forward networks  Positional encoding Classification  Flatten transformer output  Pass through linear layers with a learnable residual path using Equation (11)  Apply SoftMax activation for multi-class classification using Equation (12)  Use focal loss to address class imbalance using Equation (13)  Optimize using the Adam optimizer Return—Detected sleep disorder class for each epoch

Let $X^{EEG}\in R^{T\times C}$ represent an input EEG epoch with *T* time steps and C channels. The 1D-CNN projects each modality into a latent feature space $F^{m}\in R^{L\times d}$, where L is the reduced sequence length and d is the feature dimension. For the transformer encoder, the query, key, and value matrices are defined as $Q,\;K,\;V\in R^{L\times d}$, producing attention weights $A\in R^{L\times L}$. The gated fused representation is denoted as $F_{fused}\in R^{L\times d}$, which is processed by the transformer to obtain hidden states $h\in R^{L\times d}$. Finally, the classifier outputs probability scores $y\in R^{K}$, where K represents the number of sleep stages. The below Table 3 represents the notations summarizing symbols, descriptions, and dimensions used in proposed model.

## 5. Results and Discussion

The developed NRGAMTE model was implemented in a Python 3.7 environment on a system configured with an i5 processor, 8 GB RAM, and Windows 10 (64-bit). The parameters of the NRGAMTE model are listed in Table 4. The parameters were selected through a grid search to achieve the best balance between accuracy, robustness, and computational efficacy.

The model was trained with these chosen parameters. A maximum of 100 epochs with early stopping was employed to ensure convergence without overfitting; training for more epochs risks overfitting, while fewer epochs may lead to underfitting. A batch size of 64 was used to provide stable gradient estimation and effective GPU utilization. Larger batch sizes accelerate training but reduce generalization, whereas smaller batches introduce gradient noise and a slow convergence. A learning rate of 0.001 with the Adam optimizer was used for a stable and effective convergence; higher rates may cause divergence, while lower rates slow training and risk of local minima.

Focal loss was employed to handle class imbalance. The embedding size was fixed at 128, providing sufficient representational capacity without excessive complexity. Larger embeddings can capture richer patterns but increase the risk of overfitting and memory overhead, while smaller embeddings lose discriminative features. The 1D-CNN kernel size was set to three with a stride of 1 to capture local temporal dependencies while preserving sequence granularity. Larger kernels capture a broader context but blur the fine details, whereas smaller kernels increase the computational load.

The transformer backbone was configured with four layers and eight attention heads, which consistently obtained the best validation performance. Increasing either the depth or the number of heads raised the computational costs, while reducing them weakened the ability to model long-term dependencies. Dropout was fixed at 0.2 to regularize the model; a higher dropout adversely affects learning stability, whereas a lower dropout increases overfitting. Finally, sigmoid activation was applied in the forget gate to provide smooth gating across modalities, and a 30-s window length was used to balance the temporal resolution. Large windows risk mixing multiple sleep transitions, while shorter windows fail to capture low-frequency content.

### 5.1. Performance Metrics

- *Accuracy*—the ratio of correctly predicted samples by the model, as given in Equation (15):

(15) $$Accuracy=\frac{TP+TN}{TP+TN+FP+FN}$$

- *Precision*—the ratio of predicted positive instances that are truly positive, as in Equation (16):

(16)
P
r
e
c
i
s
i
o
n=
T
P
T
P+
F
P

- *Recall*—also called sensitivity, the ratio of actual positive instances correctly identified as positive, as in Equation (17):

(17)
R
e
c
a
l
l=
T
P
T
P+
F
N

- *F1-score*—the harmonic mean of precision and recall, which offers a comprehensive validation of the precision and recall ability of the method as in Equation (18):

(18)
F
1-
s
c
o
r
e=
2×
P
r
e
c
i
s
i
o
n×
R
e
c
a
l
l
P
r
e
c
i
s
i
o
n+
R
e
c
a
l
l

- *Specificity*—the ratio of true negative instances correctly identified as negative, as in Equation (19):

(19)
S
p
e
c
i
f
i
c
t
y=
T
N
T
N+
F
P

In these Equations (15)–(19), TP (True Positives) are samples that are actually positive and predicted as positive; TN (True Negatives) are samples that are actually negative and predicted as negative; FP (False Positives) are samples that are actually negative but predicted as positive; and FN (False Negatives) are samples that are actually positive but predicted as negative.

Table 5 presents the per-class performance analysis for the CAP Sleep database and Sleep-EDF expanded dataset. To evaluate the robustness in class-imbalanced scenarios, per-class metrics including precision, recall, F1-score, and specificity were reported for six sleep disorder classes and healthy subjects in the CAP dataset, and for five of the sleep disorder classes in the Sleep-EDF expanded dataset. From Table 5, the model demonstrates consistent performance across both majority and minority classes, with F1-scores for both datasets. This validates the model’s ability to generalize to imbalanced data.

Table 6 presents a comparative evaluation of single- and multimodal physiological signals (EEG, EMG, and EOG) on the CAP Sleep database and the Sleep-EDF expanded dataset. Each modality and their combinations were evaluated using accuracy, precision, recall, F1-score, and specificity. The results show that the developed NRGAMTE achieves a higher performance on multimodal data compared with single- or bi-modal inputs. Among individual modalities, EOG and EMG were less accurate but contributed valuable information for eye movement and muscle-activity-related disorders. While single modalities provide useful information, they provide a limited perspective of sleep behavior, leading to reduced generalizability and the misclassification of overlapping disorders. These findings highlight the necessity of a multimodal, attention-driven fusion model for accurate and robust sleep disorder detection. By integrating EEG, EMG, and EOG through the proposed fusion strategy, NRGAMTE demonstrates improved accuracy and generalization capability.

Table 7 and Table 8 show the evaluation of single modalities and multimodal combinations after incorporating MRGCAF. The results confirm that the NRGAMTE model effectively improves detection accuracy, sensitivity, and robustness when all three modalities are fused using the developed modality-wise residual gated attention fusion mechanism. The model outperforms conventional approaches by modeling cross-modal interactions and preserving significant signal-specific characteristics, making it suitable for real-world sleep disorder detection.

To validate the effectiveness of each fusion component in the developed MRGCAF module, a deep ablation analysis was conducted. Table 9 compares six different configurations: without residual path, standard (fixed) residual path, proposed learnable residual path, gated attention without any residual path, gated attention with a standard (fixed) residual path, and gated attention with a learnable residual path. The results demonstrate that the learnable residual path consistently improves all performance metrics, highlighting its significance in enhancing modality fusion and overall accuracy.

Table 10 presents an ablation analysis showing that both MRGCAF and the transformer are crucial for the proposed model. The baseline 1D-CNN extracts the local and temporal features, while incorporating MRGCAF further enhances performance by employing cross-modality forget gates and learnable residual paths to suppress redundant activations and preserve modality-specific discriminative features. Including only the transformer improves the sequence modeling through multi-head self-attention and positional embeddings, capturing the long-range temporal dependencies but without addressing cross-modal noise. The complete NRGAMTE combines both modules: the gated fusion provides stable, noise-resistant embeddings, which are passed to the transformer for global temporal modeling. This combination reduces information loss, stabilizes gradient flow, and enhances feature separability.

Table 11 presents performance comparisons with existing DL models such as Recurrent Neural Network (RNN), Long Short-Term Memory (LSTM), Gated Recurrent Unit (GRU), Vision Transformer (ViT), and Multimodal Transformer (MMT) on the CAP Sleep database and Sleep-EDF expanded dataset. The RNN and LSTM models suffer from vanishing gradient issues, lack modality awareness, and have limited training efficiency. ViT, originally designed for images, lacks the modality-specialized architecture required for physiological signal fusion when applied to time-series data. MMT does not incorporate explicit gating, which limits its ability to handle redundant signals across modalities. The LGSleepNet and BiTS-SleepNet models were re-evaluated under the same experimental settings, and their results are described in Table 11. The developed NRGAMTE achieved a 99.64% accuracy on the CAP Sleep database and 94.51% on the Sleep-EDF expanded dataset. In the proposed model, 1D-CNN-based modality embeddings preserve specific patterns in EEG, EMG, and EOG. The modality-wise residual gated attention fusion dynamically filters irrelevant and noisy cross-modal interactions, while the learnable residual path ensures that essential signal features are retained. The multimodal transformer captures long-term dependencies more effectively than traditional algorithms.

Table 12 presents the evaluation of the proposed NRGAMTE under varying K-fold cross-validation settings (K = 2 to 7) on the CAP Sleep database and Sleep-EDF expanded dataset. K-fold validation was used to analyze the generalization ability and robustness of the model across different data splits. The data were divided into K equal parts, with K-I folds used for training and one fold for testing. This process was repeated K times so that each data point was used as a test sample. Because sleep data are highly heterogeneous across subjects, K-fold cross-validation ensures the model does not overfit to a specific subset and maintains a high performance across varied sleep patterns and disorders. The results show that the proposed NRGAMTE is robust, generalizable, and resistant to overfitting, performing consistently across fold settings. Peak performance at K = 5 validates it as the optimal configuration for training and evaluating DL models on the multimodal sleep disorder dataset.

Table 13 presents the computational performance and robustness of the developed NRGAMTE compared with other DL-based algorithms. The metrics include *p*-values from ANOVA tests (to evaluate statistical significance), Memory Usage (MB), training time per epoch (seconds), and inference time per sample (seconds). The NRGAMTE model improves detection accuracy, reduces training and inference time, and maintains efficiency in highly complex multimodal settings. The MRGCAF mechanism filters redundant features, while the learnable residual path stabilizes training and preserves significant signals. Additionally, the multimodal transformer encoder effectively captures long-range temporal dependencies.

### 5.2. Cross-Dataset Generalization Analysis

To evaluate the robustness and generalization, cross-dataset experiments were conducted. The proposed NRGAMTE was trained on the CAP Sleep database and tested on the Sleep-EDF expanded dataset, and vice versa. Because CAP annotations differ from the standard AASM staging used in Sleep-EDF, the classifier layer was re-initialized and retrained on Sleep-EDF. This approach allows temporal dynamics learned from CAP to transfer while maintaining compatibility with the Sleep-EDF dataset. Table 14 reports the results, demonstrating how well the model generalizes to unseen data distributions.

### 5.3. Cross-Subject Validation

To evaluate the generalizability across individuals, cross-subject validation was performed using the Leave-One-Subject-Out (LOSO) approach on the CAP Sleep database and Sleep-EDF expanded dataset (Table 15). In each fold, data from one subject were used for testing while the model was trained on the remaining subjects. This ensures that the NRGAMTE model is evaluated on completely unseen individuals, directly addressing inter-subject variability.

To validate that the improvements in the NRGAMTE are not due to random chance, paired *t*-tests were conducted between the NRGAMTE and baseline models (MMT and ViT) on the CAP and Sleep-EDF expanded dataset (Table 16). Each model was run across 10 random seeds to ensure robustness. The *p*-values of the accuracy and F1-score were <0.05, indicating that the performance gains are statistically significant.

To validate the performance of the proposed MRGCAF fusion strategy, considered three commonly used fusion mechanisms, early fusion, direct concatenation of modality features, late fusion, independent classifiers for each modality with SoftMax score averaging, and tensor fusion, the outer product of modality features, followed by projection layers. These models were trained under the same settings using the CAP and Sleep-EDF expanded dataset. As depicted in Table 17, the MRGCAF outperformed all alternatives, especially in the F1-score and minority-class recall. This shows that gated cross-modal interaction, combined with a learnable residual path, offers a superior strategy for physiological signals.

### 5.4. Model Diagnostic Analysis

**Figure 5:**
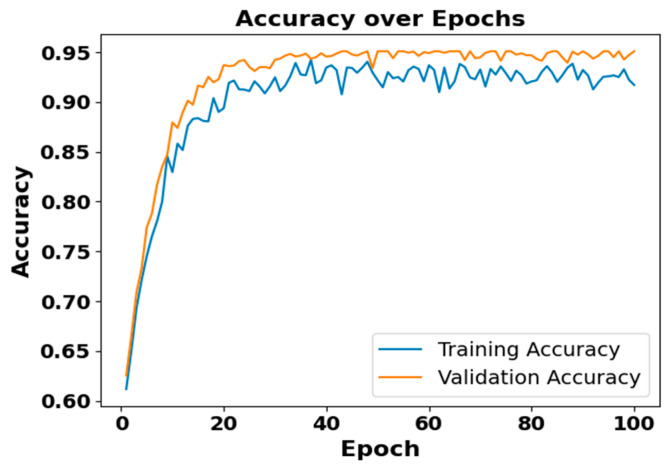
Accuracy vs. Epochs for Sleep-EDF expanded dataset.

**Figure 6:**
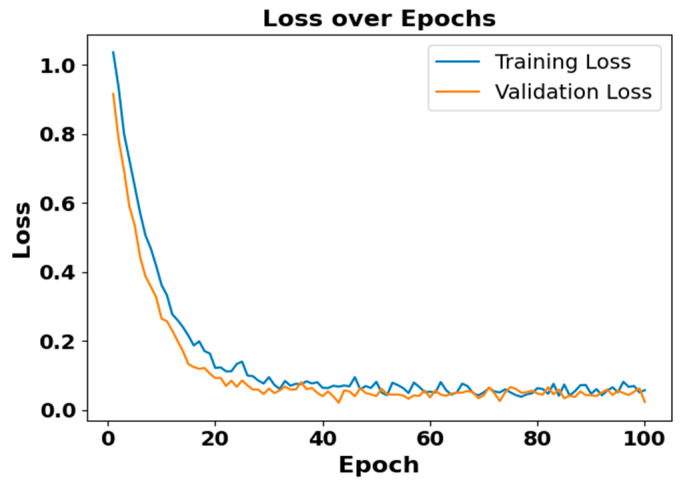
Loss vs. epochs for Sleep-EDF dataset.

**Figure 7:**
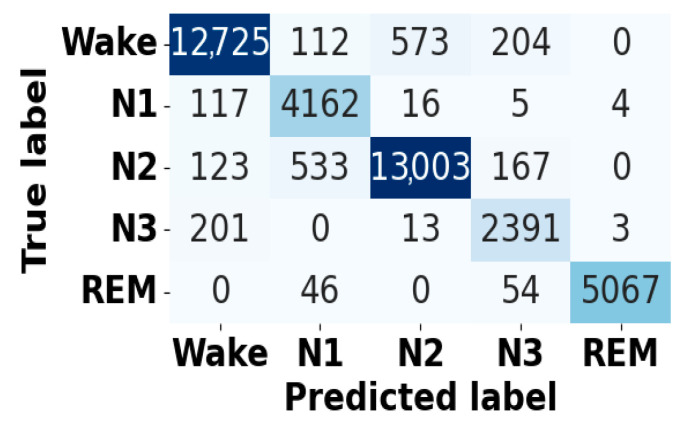
Confusion matrix for Sleep-EDF dataset.

**Figure 8:**
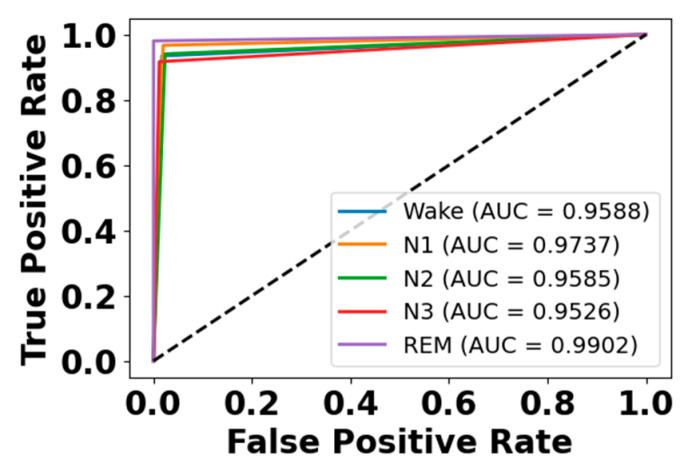
ROC curve for Sleep-EDF dataset.

Figure 5 and Figure 6 present the accuracy vs. epoch and loss vs. epoch graphs for the Sleep-EDF expanded dataset, respectively. Figure 7 represents the confusion matrix, and Figure 8 illustrates the ROC Curve for the same dataset. The confusion matrix provides a detailed explanation of the model’s predictions across all sleep disorder classes, showing how many instances were correctly classified and where misclassification occurred. This is essential for evaluating the class-specific performance and validating the model’s reliability in identifying overlapping disorders with similar features. The ROC curve validates the trade-off between the true positive rate and false positive rate across thresholds. This is essential for calculating the discrimination power for each disorder class and comparing performance across different classes.

**Figure 9:**
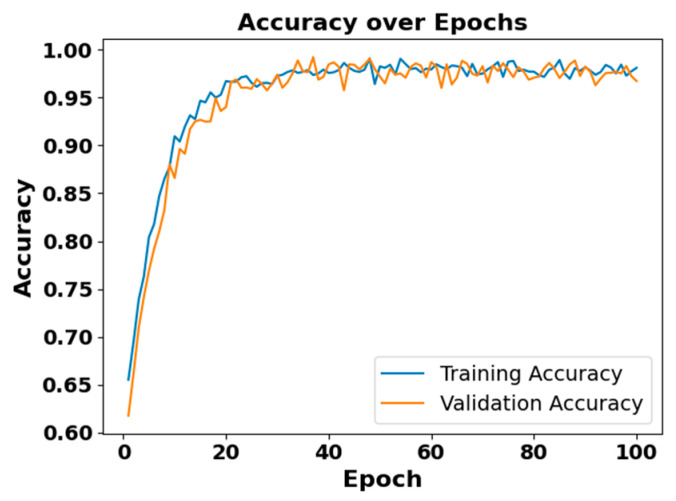
Accuracy vs. epoch for EEG modality on CAP Sleep database.

**Figure 10:**
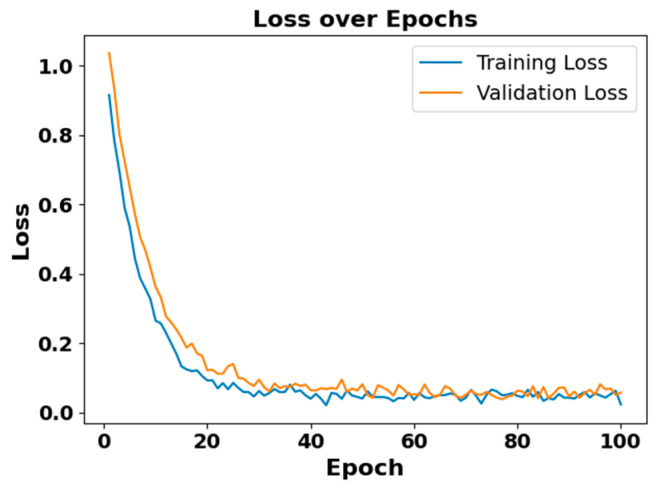
Loss vs. epoch for EEG modality on CAP Sleep database.

**Figure 11:**
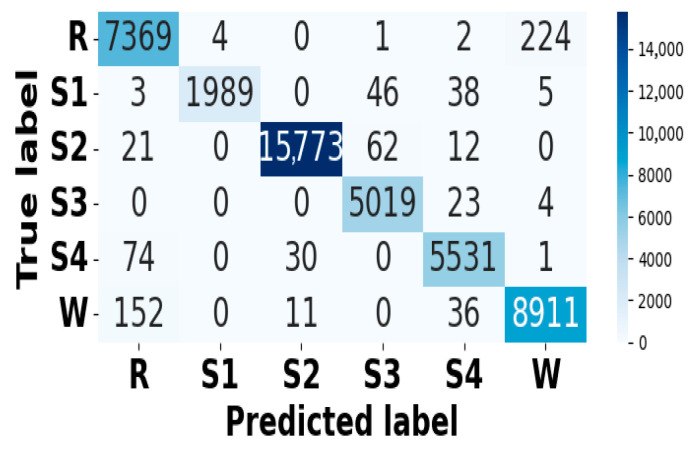
Confusion matrix for EEG modality on CAP Sleep database.

**Figure 12:**
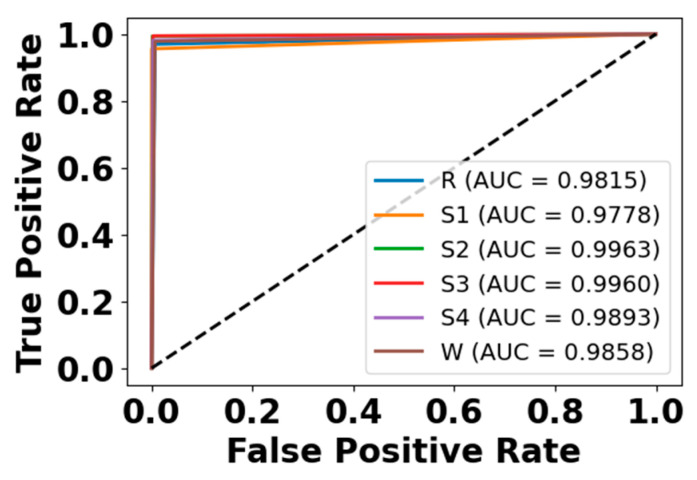
ROC curve for EEG modality on CAP Sleep database.

**Figure 13:**
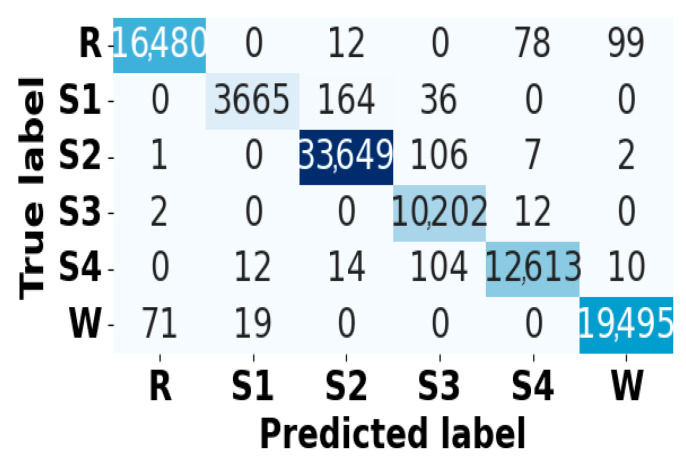
Confusion matrix for EOG modality on CAP Sleep database.

**Figure 14:**
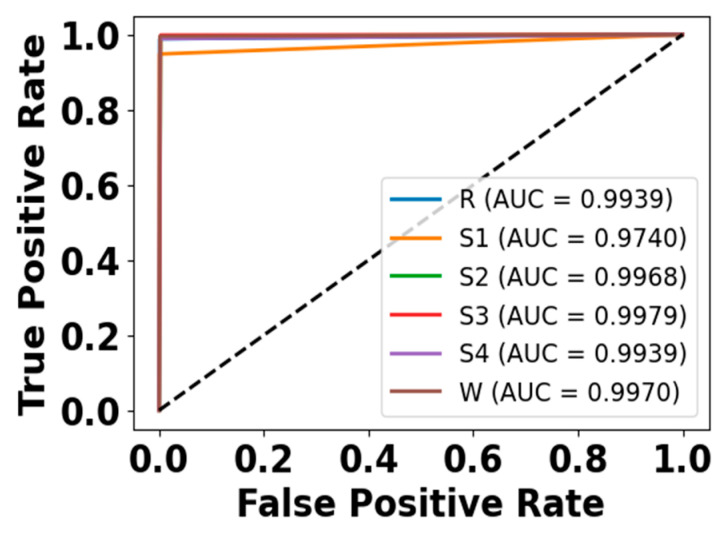
ROC curve for EOG modality on CAP Sleep database.

**Figure 15:**
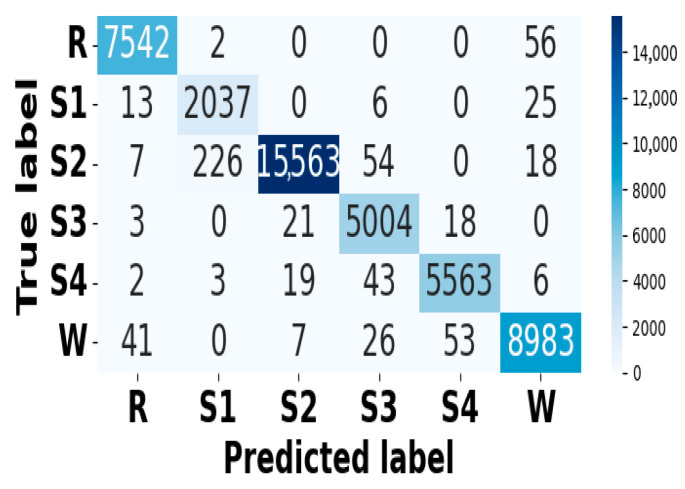
Confusion matrix for EMG modality on CAP Sleep database.

**Figure 16:**
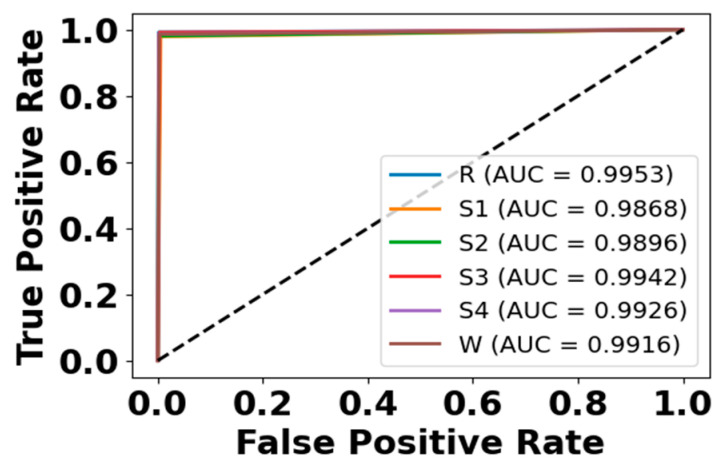
ROC curve for EMG modality on CAP Sleep database.

Figure 9 and Figure 10 present accuracy vs. epoch and loss vs. epoch graphs for the EOG modality on the CAP Sleep database. Figure 11 shows the confusion matrix, while Figure 12 represents the ROC Curve for the same modality database. Figure 13 and Figure 14 represent the confusion matrix and ROC Curve for the EEG modality on the CAP Sleep database. Figure 15 and Figure 16 present the confusion matrix and ROC Curve for the EMG modality on the CAP Sleep database.

### 5.5. Comparative Analysis

This section compares the performance of the developed NRGAMTE model with the existing algorithms on the CAP Sleep database and Sleep-EDF expanded dataset. The comparison is performed using both single-modality and multimodality signals.

#### 5.5.1. Single-Modality-Based Comparison

Table 18 presents a comparison of the developed NRGAMTE model with existing algorithms using single modalities such as 1D-CNN [36], EbagT [37], kNN [38], 1D-CNN-CAM [39], LSTM + CNN [40], MWTCNNNet [41], and WASR-LCNN [42] on the CAP Sleep database and Sleep-EDF expanded dataset.

#### 5.5.2. Multi-Modality-Based Comparison

Table 19 presents a comparison of the developed NRGAMTE model with existing algorithms using multiple modalities such as CareSleepNet [43], MM-DMS-Distributed CNN + PT Shallow [44], and MML-DMS [45] on the CAP Sleep database and Sleep-EDF expanded dataset. The developed NRGAMTE improves the detection accuracy, minimizes the computational time in training and inference, and demonstrates statistical improvements in the detection outcomes. The MRGCAF mechanism filters redundant features, while the learnable residual path stabilizes training and preserves significant signals. Additionally, the multimodal transformer encoder captures the long-range temporal dependencies.

### 5.6. Discussion

The proposed NRGAMTE model introduces a multimodal transformer improved with the MRGCAF mechanism, which addresses the key challenges in sleep disorder detection such as inter-subject variability, overlapping disorder symptoms, and the drawbacks of unimodal algorithms. By combining EEG, EOG, and EMG signals, the model leverages complementary physiological information, enabling robust performance across both the CAP Sleep and Sleep-EDF expanded dataset.

A major contribution of this research is the learnable residual path integrated with the gating mechanism in MRGCAF. Conventional recurrent models such as Bi-GRU have demonstrated that gating allows the selective retention of long-term temporal dependencies while filtering out irrelevant data. For example, smoothing and matrix-decomposition-based stacked Bi-GRU models in downtime forecasting rely on such gates to stabilize long-horizon predictions. Although the proposed model does not rely on Bi-GRU, the developed gating mechanism plays an analogous role by filtering noisy cross-modal interactions and preserving critical modality-specific features. This mechanism acts as a selective filter, retaining long-range dependencies while discarding irrelevant fluctuations, thereby providing the multimodal transformer with stable and informative embeddings for improved sleep disorder detection.

This ensures that the multimodal transformer receives stable and informative embeddings, thereby enhancing its ability to capture long-range dependencies without short-term fluctuations or information loss. The learnable residual path further enhances this process by adaptively reweighting retained features, improving gradient flow, and enhancing interpretability. This architecture enables the method to obtain superior accuracy while maintaining robustness across minority classes and inter-subject variability. The ablation analysis shows that the learnable residual path consistently enhances the detection performance compared to fixed or non-residual gating strategies. This research determines that gating-enhanced attention acts as a scalable alternative to recurrent decomposition models for long-sequence modeling. Unlike Bi-GRU-based algorithms that depend heavily on recurrence, the transformer-based model allows parallelized computation and better generalization, while the gating unit ensures that stable and relevant data contribute to long-range temporal modeling. This balance shows that NRGAMTE achieves an accuracy, robustness, and interpretability suitable for real-world sleep disorder detection process.

### 5.7. Research Implication

The developed NRGAMTE model introduces a novel and effective framework for sleep disorder detection using multimodal physiological signals and attention-based fusion mechanisms. This model not only improves detection performance but also provides valuable insights into building scalable and interpretable models for physiological signals. The primary implications are summarized below:

- Enhanced detection accuracy in multimodal sleep analysis—by combining EEG, EMG, and EOG with modality-wise gated attention, the NRGAMTE model effectively enhances the accuracy and reliability of sleep disorder detection. This enhancement supports the development of more effective clinical decision-support systems.
- Efficient feature elimination via gated attention mechanism—the incorporation of cross-modality forget gates with learnable residual paths enables the model to filter irrelevant and noisy modality-specific features, and improves generalization across subjects.
- Transformer Viability—by adapting transformer architecture for multimodal signals, the model validates the scalability and applicability of attention-based models in complex time-series learning tasks involving temporal and cross-modal dependencies.
- Trade-off between performance and computational efficacy—the NRGAMTE achieves superior detection while minimizing the training and inference time, outperforming the existing baselines in both accuracy and runtime efficiency.

Although the overall accuracy improvement in the proposed NRGAMTE (94.51%) over EEG only (93.76%) or multimodal fusion without MRGCAF (94.24%) on Sleep-EDF may appear marginal, the added complexity of MRGCAF is justified. MRGCAF achieves a consistently higher precision, recall, F1-score, and specificity across classes, especially in minority stages such as N1 and REM, where EEG-only models show performance drops. Cross-dataset generalization and cross-subject validation further confirm that NRGAMTE with MRGCAF offers a stable and robust performance across heterogeneous populations, reducing the sensitivity to inter-subject variability. Moreover, the learnable residual gating mechanism improves interpretability by filtering redundant cross-modal interactions while retaining essential modality-specific data, which is difficult to achieve with simple concatenation or early fusion. Thus, even modest aggregate accuracy gains reflect meaningful improvements in the class-level balance, robustness, and clinical reliability.

### 5.8. Limitations

Despite its efficiency, the proposed NRGAMTE model has several technical constraints. Its reliance on multimodal inputs (EEG, EOG, and EMG) limits its applicability in scenarios with noisy data. The transformer-based fusion with gated residual attention increases the computational cost and memory usage, making deployment on low power challenging. Furthermore, despite cross-dataset validation, generalization to additional modalities such as SpO_2_, and airflow remain untested. Finally, interpretability is limited to attention-based gating, and the integration with more advanced Explainable Artificial Intelligence (XAI) model is required for clinical transparency.

## 6. Conclusions

In this manuscript, we developed the DL framework named NRGAMTE for enhancing sleep disorder detection using multimodal physiological signals, including EEG, EMG, and EOG. The developed modal incorporated a modality-wise residual gated attention fusion mechanism, which enabled selective and interpretable feature fusion while preserving essential modality-specific data through a learnable residual path. Each signal stream was embedded using 1D-CNN, and temporal dependencies were captured through a multimodal transformer encoder.

The NRGAMTE model efficiently addresses the drawbacks of single-modality and sequential architectures. The experimental results on the CAP Sleep database and the Sleep-EDF expanded dataset show that NRGAMTE achieves superior accuracy, reduced inference time, and enhanced statistical significance compared with traditional algorithms. These findings highlight its promise as a scalable and effective approach for automatic sleep disorder detection.

Cross-subject validation demonstrates that the NRGAMTE model generalizes effectively across unseen individuals, supporting its applicability in real-world deployments. Moreover, the NRGAMTE model demonstrates resilience to class imbalance, supported by the use of focal loss and minority-class performance metrics.

### Future Work

As future work, the focus will be on extending the NRGAMTE model to incorporate additional physiological signals, such as SpO_2_ and airflow, to enable broader disorder coverage. Cross-data generalization and personalization strategies will also be explored to further enhance robustness across diverse populations.

## Figures and Tables

**Table 1:** Comparison of existing sleep disorder detection methods with the proposed NRGAMTE model in terms of dataset, modality, methodology, and performance.

Methods	Dataset	Modality	Accuracy (%)	Techniques Used	Performance Metrics
1D-CNN [36]	CAP Sleep	EEG	90.46	1D-CNN	Accuracy, Precision, Recall, F1-score
EbagT [37]	CAP Sleep	ECG	98.00	Ensemble Bag of Trees	Accuracy
kNN [38]	CAP Sleep	EEG	79.14	K-Nearest Neighbors	Accuracy, Precision, Recall, F1-score
1D-CNN-CAM [39]	CAP Sleep	EEG	90.31	CNN + Attention	Accuracy, F1-score, Specificity
LSTM + CNN [40]	CAP Sleep	EEG	91.45	LSTM + CNN	Accuracy
MWTCNNNet [41]	CAP Sleep	EEG	98.90	Morlet Wavelet + CNN	Accuracy, Recall, Specificity
WASR-LCNN [42]	Sleep-EDF expanded	EEG	87.60	Wavelet + Light-weight CNN	Accuracy, F1-score
CareSleepNet [43]	Sleep-EDF expanded	EEG + EOG	85.10	CNN + Transformer	Accuracy, F1-score
MM-DMS-Distributed CNN + PT Shallow [44]	CAP Sleep	EEG + ECG + EMG	95.43	Shallow Neural Networks	Accuracy
MML-DMS [45]	CAP Sleep	EEG + ECG + EMG	99.09	Multimodal Multilabel Neural Network	Accuracy
Proposed NRGAMTE	CAP Sleep	EEG + EOG + EMG	99.64	1D CNN + Multimodal Transformer + MRGCAF	Accuracy, Precision, Recall, F1-score, Specificity
Proposed NRGAMTE	Sleep-EDF expanded	EEG + EOG + EMG	94.51	1D CNN + Multimodal Transformer + MRGCAF	Accuracy, Precision, Recall, F1-score, Specificity

**Table 2:** Dataset description.

Sleep Stages	Healthy	Sleep Disorder					Total
		Insomnia	Narcolepsy	PLM	RBD	NFLE	
Wake	451	3804	1305	1363	4436	3282	14,641
S1	280	223	301	284	844	1165	3097
S2	2172	2456	1708	2845	7123	11,317	27,621
S3	573	670	476	988	2820	3111	8638
S4	1184	415	568	955	2331	4362	9815
REM	1409	986	1258	1340	3364	5205	13,562
Total	6069	8554	5616	7775	20,918	28,442	77,374

**Table 3:** Table of notations summarizing symbols, descriptions, and the dimensions used in proposed NRGAMTE model.

Symbol	Description	Dimension
X E E G, X E O G, X E M G	Input signal epoch	R T× C
F E E G, F E O G, F E M G	CNN extracted feature vectors	R L× d
Q, K, V	Query, Key, Value matrices (transformer input)	R L× d
A	Attention weight matrix	R L× L
F f u s e d	Gated multimodal fused feature	R L× d
h	Hidden representation after transformer	R L× d
y	Output prediction (sleep stage class)	$R^{K}$, where K=number of classes

**Table 4:** Parameter setting for the developed NRGAMTE model.

Parameters	Value	Description
Epochs	100	Number of training cycles
Batch size	64	Number of samples per training batch
Learning rate	0.001	Initial step size for weight updates
Optimizer	Adam	Adaptive moment estimation optimizer
Loss function	Focal loss	Handles class imbalance
Embedding size	128	Dimension of shared modality embedding
1D-CNN kernel size	3	Receptive field of CNN for every modality
1D-CNN stride	1	Shift size in convolution
Transformer layers	4	Number of encoder blocks in multimodal transformer
Number of attention heads	8	Number of cross-gated attention per transformer block
Dropout rate	0.2	Regularization factor for preventing overfitting
Forget gate activation	Sigmoid	Activation function used in the gating mechanism
Segment window size	30 s	Fixed window size

**Table 5:** Per-class performance metrics analysis for CAP Sleep database and Sleep-EDF expanded dataset.

Classes	Precision (%)	Recall (%)	F1-Score (%)	Specificity (%)
CAP Sleep Database				
NFLE	99.70	99.80	99.75	99.83
PLM	98.80	98.91	98.85	98.94
RDB	98.71	98.66	98.68	98.79
Insomnia	99.04	98.77	98.90	99.02
Narcolepsy	98.92	99.13	99.02	98.96
Healthy	99.81	99.76	99.78	99.85
Sleep-EDF expanded dataset				
Wake (W)	95.81	95.36	95.58	96.03
N1	91.04	89.91	90.47	92.22
N2	94.28	95.13	94.70	93.89
N3	94.91	94.67	94.79	95.18
REM	95.02	94.68	94.85	95.26

**Table 6:** Evaluation of single and multimodal physiological signal modalities such as EEG, EMG, and EOG.

Modalities	Accuracy (%)	Precision (%)	Recall (%)	F1-Score (%)	Specificity (%)
CAP Sleep database					
EEG	99.23	99.07	99.31	99.18	99.25
EOG	98.35	98.02	99.08	98.54	99.02
EMG	98.57	98.21	98.76	98.48	98.82
EEG + EOG	97.64	97.32	97.71	97.51	97.75
EEG + EMG	97.72	97.53	97.65	97.58	97.68
EOG + EMG	98.05	97.89	97.66	97.77	97.74
EEG + EOG + EMG (NRGAMTE)	99.64	99.32	99.55	99.43	99.71
Sleep-EDF Expanded					
EEG	93.76	93.41	94.62	94.01	95.05
EOG	93.14	92.87	92.52	92.69	93.44
EMG	93.42	93.16	93.88	93.51	93.65
EEG + EOG	90.25	90.02	92.44	91.21	93.18
EEG + EMG	90.41	90.25	92.17	91.19	92.67
EOG + EMG	90.53	90.37	92.35	91.34	92.55
EEG + EOG + EMG (NRGAMTE)	94.51	94.32	95.03	94.67	94.95

**Table 7:** Evaluation of Single Physiological Signal modalities like EEG, EMG, and EOG before and after using MRGCAF.

Modalities	Accuracy (%)	Precision (%)	Recall (%)	F1-Score (%)	Specificity (%)
CAP Sleep database					
EEG with MRGCAF	99.23	99.07	99.31	99.18	99.25
EEG without MRGCAF	98.46	98.29	98.12	98.20	98.51
EOG with MRGCAF	98.35	98.02	99.08	98.54	99.02
EOG without MRGCAF	98.24	98.02	97.87	97.95	98.46
EMG with MRGCAF	98.57	98.21	98.76	98.48	98.82
EMG without MRGCAF	98.22	98.04	97.83	97.95	98.35
Sleep-EDF Expanded					
EEG with MRGCAFL	93.76	93.41	94.62	94.01	95.05
EEG without MRGCAFL	93.41	93.15	92.89	92.98	93.55
EOG with MRGCAFL	93.14	92.87	92.52	92.69	93.44
EOG without MRGCAFL	92.87	92.65	92.47	92.54	93.16
EMG with MRGCAFL	93.42	93.16	93.88	93.51	93.65
EMG without MRGCAFL	93.67	93.42	93.22	93.36	93.97

**Table 8:** Evaluation of Multimodal Physiological Signal modalities such as EEG, EMG, and EOG, before and after using MRGCAF.

Modalities	Accuracy (%)	Precision (%)	Recall (%)	F1-Score (%)	Specificity (%)
CAP Sleep database					
EEG + EOG with MRGCAFL	97.64	97.32	97.71	97.51	97.75
EEG + EOG without MRGCAFL	97.42	97.25	97.06	97.15	97.56
EEG + EMG with MRGCAFL	97.72	97.53	97.65	97.58	97.68
EEG + EMG without MRGCAFL	97.55	97.32	97.18	97.25	97.72
EOG + EMG with MRGCAFL	98.05	97.89	97.66	97.77	97.74
EOG + EMG without MRGCAFL	97.66	97.53	97.21	97.34	97.82
EEG + EOG + EMG with MRGCAFL	99.64	99.32	99.55	99.43	99.71
EEG + EOG + EMG without MRGCAFL	99.42	99.28	98.92	99.07	99.55
Sleep-EDF Expanded					
EEG + EOG with MRGCAFL	90.25	90.02	92.44	91.21	93.18
EEG + EOG without MRGCAFL	90.03	89.67	92.15	91.06	92.79
EEG + EMG with MRGCAFL	90.41	90.25	92.17	91.19	92.67
EEG + EMG without MRGCAFL	90.15	90.08	91.87	90.85	91.23
EOG + EMG with MRGCAFL	90.53	90.37	92.35	91.34	92.55
EOG + EMG without MRGCAFL	90.34	90.14	92.09	91.21	91.76
EEG + EOG + EMG with MRGCAFL	94.51	94.32	95.03	94.67	94.95
EEG + EOG + EMG without MRGCAFL	94.24	94.18	94.64	94.43	94.35

**Table 9:** Evaluation of NRGAMTE model with various residual paths on CAP Sleep database and Sleep-EDF expanded dataset to assess the contribution of the learnable residual path in the fusion process.

Models	Accuracy (%)	Precision (%)	Recall (%)	F1-Score (%)	Specificity (%)
CAP Sleep database					
Without residual path	98.84	98.51	98.66	98.58	98.73
Standard (fixed) residual path	99.12	98.95	99.07	99.01	99.20
Proposed Learnable residual path	99.64	99.32	99.55	99.43	99.71
Gating only (no residual path)	98.84	98.51	98.66	98.58	98.73
Gating + standard residual path	99.12	98.95	99.07	99.01	99.20
Proposed Gating + Learnable residual path	99.64	99.32	99.55	99.43	99.71
Sleep-EDF expanded dataset					
Without residual path	93.78	93.41	94.15	93.77	94.06
Standard (fixed) residual path	94.12	93.88	94.47	94.17	94.35
Proposed Learnable residual path	94.51	94.32	95.03	94.67	94.95
Gating only (no residual path)	93.78	93.41	94.15	93.77	94.06
Gating + standard residual path	94.12	93.88	94.47	94.17	94.35
Proposed Gating + Learnable residual path	94.51	94.32	95.03	94.67	94.95

**Table 10:** Ablation analysis determines contributions of MRGCAF and transformer modules to NRGAMTE performance.

Methods	Accuracy (%)	Precision (%)	Recall (%)	F1-Score (%)	Specificity (%)
CAP Sleep database					
1D-CNN without MRGCAF and Transformer	98.84	98.50	98.61	98.55	98.70
1D-CNN + MRGCAF without transformer	99.10	98.72	99.00	98.85	99.05
1D-CNN + transformer without MRGCAF	99.32	99.00	99.20	99.10	99.25
Proposed NRGAMTE (1D-CNN + MRGCAF + Transformer)	99.64	99.32	99.55	99.43	99.71
**Sleep-EDF expanded dataset**					
1D-CNN without MRGCAF and Transformer	93.78	93.40	94.00	93.69	94.05
1D-CNN + MRGCAF without transformer	94.12	93.82	94.35	94.08	94.28
1D-CNN + transformer without MRGCAF	94.03	93.75	94.25	93.95	94.20
Proposed NRGAMTE (1D-CNN + MRGCAF + Transformer)	94.51	94.32	95.03	94.67	94.95

**Table 11:** Evaluation of developed NRGAMTE over traditional algorithms on the CAP Sleep database and the Sleep-EDF expanded dataset.

Methods	Accuracy (%)	Precision (%)	Recall (%)	F1-Score (%)	Specificity (%)
CAP Sleep database					
RNN	98.07	97.89	97.56	97.74	98.42
LSTM	98.43	98.26	98.07	98.16	98.67
GRU	98.76	98.54	98.32	98.43	98.92
ViT	99.04	98.87	98.63	98.74	99.27
LGSleepNet	99.10	98.95	98.77	98.85	99.15
BiTS-SleepNet	99.23	99.02	98.82	98.91	99.34
MMT	99.32	99.16	98.89	98.97	99.53
Proposed NRGAMTE	99.64	99.32	99.55	99.43	99.71
Sleep-EDF expanded data					
RNN	92.76	92.65	93.41	93.77	93.57
LSTM	93.09	92.87	93.68	93.42	93.86
GRU	93.43	93.02	94.03	93.75	94.15
ViT	93.76	93.46	94.43	93.79	94.47
LGSleepNet	93.84	93.52	94.27	93.89	93.97
BiTS-SleepNet	93.95	93.72	94.15	93.93	94.12
MMT	94.03	93.85	94.76	93.98	94.63
Proposed NRGAMTE	94.51	94.32	95.03	94.67	94.95

**Table 12:** Evaluation of NRGAMTE under different K-fold cross-validation on the CAP Sleep database and the Sleep-EDF expanded dataset.

K-Fold Values	Accuracy (%)	Precision (%)	Recall (%)	F1-Score (%)	Specificity (%)
CAP Sleep database					
K = 2	98.02	98.43	98.67	98.83	99.17
K = 3	98.21	98.78	99.07	99.03	99.33
K = 4	98.45	99.02	99.21	99.21	99.52
K = 5	99.64	99.32	99.55	99.43	99.71
K = 6	98.37	98.83	98.79	98.76	98.79
K = 7	98.21	98.65	98.25	98.54	98.43
Sleep-EDF data					
K = 2	94.04	93.84	94.37	94.28	94.26
K = 3	94.22	94.05	94.64	94.44	94.48
K = 4	94.36	94.18	94.86	94.51	94.87
K = 5	94.51	94.32	95.03	94.67	94.95
K = 6	94.28	94.15	94.69	94.37	94.64
K = 7	94.17	94.02	94.38	94.02	94.37

**Table 13:** Evaluation of computational performance and statistical analysis on the CAP Sleep database and Sleep-EDF expanded data.

Methods	*p*-Value (ANOVA Test)	Memory Usage (MB)	Training Time per Epoch (s)	Inference Time (s)
CAP Sleep database				
RNN	0.047	378	125	115
LSTM	0.042	403	99	98
GRU	0.033	436	92	91
ViT	0.028	479	84	82
MMT	0.017	521	79	73
Proposed NRGAMTE	0.004	567	71	65
Sleep-EDF expanded data				
RNN	0.051	857	120	110
LSTM	0.045	835	97	92
GRU	0.032	812	92	89
ViT	0.024	776	89	82
MMT	0.011	732	83	77
Proposed NRGAMTE	0.003	645	75	68

**Table 14:** Evaluation of cross-dataset generalization analysis using CAP Sleep database and Sleep-EDF expanded dataset.

Training Dataset	Testing Dataset	Accuracy (%)	Precision (%)	Recall (%)	F1-Score (%)	Specificity (%)
CAP Sleep database	Sleep-EDF expanded dataset	91.88	91.56	92.71	92.13	92.94
Sleep-EDF expanded dataset	CAP Sleep database	96.41	96.12	96.75	96.43	96.89

**Table 15:** Evaluation of cross-subject validation for NRGAMTE model using CAP Sleep database and Sleep-EDF expanded dataset.

Metrics	Accuracy (%)	Precision (%)	Recall (%)	F1-Score (%)	Specificity (%)
**CAP Sleep database**					
Mean (%)	97.83	97.62	97.88	97.74	98.12
Standard deviation	±0.89	±0.94	±0.91	±0.97	±0.88
**Sleep-EDF expanded dataset**					
Mean (%)	92.43	91.88	92.57	92.21	93.02
Standard deviation	±1.26	±1.32	±1.28	±1.30	±1.17

**Table 16:** Evaluation of the NRGAMTE model for statistical significance using the CAP Sleep database and Sleep-EDF expanded dataset.

Models	Accuracy (%)	CI (±)	F1-Score (%)	CI (±)	*p*-Value
**CAP Sleep database**					
ViT	99.04	±0.21	98.74	±0.26	0.031
MMT	99.32	±0.19	98.97	±0.22	0.025
Proposed NRGAMTE	99.64	±0.14	99.43	±0.18	0.004
**Sleep-EDF expanded dataset**					
ViT	93.76	±0.42	93.79	±0.38	0.029
MMT	94.03	±0.35	93.98	±0.34	0.022
Proposed NRGAMTE	94.51	±0.31	94.67	±0.28	0.003

**Table 17:** Evaluation of proposed MRGCAF fusion strategy with different fusion mechanisms using CAP Sleep database and Sleep-EDF expanded dataset.

Fusion Methods	Accuracy (%)	Precision (%)	Recall (%)	F1-Score (%)	Params (M)
CAP Sleep database					
Early Fusion	98.84	98.46	98.31	98.38	0.45
Late Fusion	98.71	98.50	98.09	98.29	0.43
Tensor Fusion	99.10	98.85	99.04	98.94	0.91
Proposed MRGCAF	99.64	99.32	99.55	99.43	0.53
Sleep-EDF expanded dataset					
Early Fusion	92.83	92.41	92.35	92.38	0.45
Late Fusion	92.57	92.10	91.91	92.00	0.43
Tensor Fusion	93.47	93.05	93.28	93.16	0.91
Proposed MRGCAF	94.51	94.32	95.03	94.67	0.53

**Table 18:** Comparative analysis of the developed NRGAMTE model on single modality with CAP Sleep database and Sleep-EDF expanded dataset.

Methods	Dataset	Modality	Accuracy (%)	Precision (%)	Recall (%)	F1-Score (%)	Specificity (%)
1D-CNN [36]	CAP Sleep database	EEG	90.46	79.22	95.73	86.70	NA
EbagT [37]		ECG	98.0	NA	NA	NA	NA
kNN [38]		EEG	79.14	79.62	78.86	79.24	NA
1D-CNN-CAM [39]		EEG	90.31	70.67	62.58	65.73	95.30
LSTM + CNN [40]		EEG	91.45	NA	NA	NA	NA
MWTCNNNet [41]		EEG	98.9	NA	99.03	NA	99.27
WASR-LCNN [42]	Sleep-EDF expanded	EEG	87.6	NA	NA	82.1	NA
Proposed NRGAMTE	CAP Sleep database	EEG	99.23	99.07	99.31	99.18	99.25
		EOG	98.35	98.02	99.08	98.54	99.02
		EMG	98.57	98.21	98.76	98.48	98.82
	Sleep-EDF expanded	EEG	93.76	93.41	94.62	94.01	95.05
		EOG	93.14	92.87	92.52	92.69	93.44
		EMG	93.42	93.16	93.88	93.51	93.65

**Table 19:** Comparative analysis of the developed NRGAMTE model on multi-modality with the CAP Sleep database and Sleep-EDF expanded dataset.

Methods	Dataset	Modality	Accuracy (%)	Precision (%)	Recall (%)	F1-Score (%)	Specificity (%)
CareSleepNet [43]	Sleep-EDF expanded	EEG + EOG	85.1	NA	NA	80.4	NA
MM-DMS-Distributed CNN + PT Shallow [44]	CAP Sleep database	EEG + ECG + EMG	95.43	NA	NA	NA	NA
MML-DMS [45]		EEG + ECG + EMG	99.09	NA	NA	NA	NA
Proposed NRGAMTE	Sleep-EDF expanded	EEG + EOG	90.25	90.02	92.44	91.21	93.18
		EEG + EMG	90.41	90.25	92.17	91.19	92.67
		EOG + EMG	90.53	90.37	92.35	91.34	92.55
		EEG + EOG + EMG (NRGAMTE)	94.51	94.32	95.03	94.67	94.95
	CAP Sleep database	EEG + EOG	97.64	97.32	97.71	97.51	97.75
		EEG + EMG	97.72	97.53	97.65	97.58	97.68
		EOG + EMG	98.05	97.89	97.66	97.77	97.74
		EEG + EOG + EMG (NRGAMTE)	99.64	99.32	99.55	99.43	99.71

## Data Availability

The original data presented in the study are openly available in the CAP Sleep database and Sleep-EDF expanded dataset at [https://physionet.org/content/capslpdb/1.0.0/ and https://www.physionet.org/content/sleep-edfx/1.0.0/]. accessed on 6 September 2025.
